# Alternative functions of Hd1 in repressing or promoting heading are determined by Ghd7 status under long-day conditions

**DOI:** 10.1038/s41598-017-05873-1

**Published:** 2017-07-14

**Authors:** Zhanyi Zhang, Wei Hu, Guojing Shen, Haiyang Liu, Yong Hu, Xiangchun Zhou, Touming Liu, Yongzhong Xing

**Affiliations:** National Key Laboratory of Crop Genetic Improvement, Hubei Collaborative Innovation Center for Grain Industry, Huazhong Agricultural University, Wuhan, 430070 China

## Abstract

Previous studies suggested that *Hd1* promoted heading under short-day conditions (SD) and delayed heading under long-day conditions (LD). However in this study, *Hd1* was demonstrated to consistently promote heading date in Zhenshan 97 (ZS97) background by upregulating *Ehd1*, *Hd3a* and *RFT1* expression under both SD and LD. While the high photoperiod sensitivity of *Hd1* was observed in Minghui 63 (MH63) background, with heading being suppressed in LD but promoted in SD. Comparative analysis of two sets of near isogenic lines of *Hd1* in MH63 and ZS97 backgrounds indicated that the alternative functions of *Hd1* in promoting or suppressing heading under LD are dependent on the previously cloned flowering repressor gene *Ghd7*. The interaction between proteins Ghd7 and Hd1 occurred through binding of the CCT domain of Ghd7 to the transcription-activating domain of Hd1, resulting in suppression of *Ehd1* and florigen gene expression. The involvement of the transcription-activating domain of Hd1 in this protein-protein interaction probably blocked or weakened its transcriptional activity. These findings suggest that *Hd1* alone essentially acts as a promoter of heading date, and the protein interaction between Ghd7 and Hd1 determines photoperiod sensitivity and integrated *Hd1-*mediated and *Ehd1-*mediated flowering pathways in rice.

## Introduction

Rice (*Oryza sativa L*.) is a facultative short-day plant. It perceives the day length and flowers faster in short-day (SD) than in long-day (LD) conditions. Rice is cultivated widely across the globe, ranging from 45° north latitude to 35° south latitude^1^. Thus, rice shows high natural variations in heading date, which influences the responses of this crop to different photoperiods and temperatures. Heading date (HD) is a crucial determinant of regional and seasonal adaptation in rice^2^. Understanding the genetic basis of heading date will aid in the design of breeding schemes for specific regions and cropping seasons and contribute to safe rice production.

Since 2000, many heading date-related QTLs have been mapped using different genetic populations and methods^3–11^. Several major heading date QTLs have been cloned to date using a map-based cloning strategy^12–19^. *Hd1*, the ortholog of *Arabidopsis CONSTANS* (*CO*), is a major gene controlling heading date in rice. *Hd1* inhibits *Hd3a* expression and delays heading date under LD and promotes *Hd3a* expression and accelerates heading date under SD^19^. *Hd3a*, the ortholog of *Arabidopsis FT*, is a rice florigen gene that is regulated by *Hd1* ^13^. *Ehd1*, which encodes a B-type response regulator, is a rice-specific heading date gene functioning upstream of *Hd3a* for which no ortholog has been identified in *Arabidopsis* ^20^. Several genes with pleiotropic effects on heading date, plant height, grain number and grain yield have been identified, such as *Ghd7*, *Ghd8*/*DTH8* and *Ghd7*.*1* ^15–18^. These genes delay heading date by suppressing *Ehd1* and increasing grain yield under LD. Two genes, *Hd6* and *Hd16*, encoding casein kinases^14, 21^, regulate heading at protein level, in which Hd6 and Hd16 phosphorylate Ghd7 and Ghd7.1 *in vitro*, respectively, and delay heading date in LD^22^. In addition, several heading date genes, such as *OsGI*, *RID1*/*OsId1*/*Ehd2*, *OsMADS51*, *Ehd3* and *HAF1*, have been isolated through reverse genetics^23–27^. This research progress contributes to our understanding of the genetic control of heading date in rice.

The molecular mechanism underlying photoperiod flowering has been well characterized in *Arabidopsis* and rice^28–32^. *Arabidopsis* is a typical long-day plant, and its photoperiodic flowering pathway is mediated by the *GI*-*CO*-*FT* pathway^28^. In contrast, two independent pathways have been identified in rice: the *OsGI*-*Hd1*-*Hd3a* pathway is conserved, sharing high similarity with the *GI*-*CO*-*FT* pathway in *Arabidopsis* ^30^, while the other pathway is mediated by *OsGI*-*Ehd1*-*Hd3a*, a unique flowering pathway in rice. The genes of *Ghd7*, *Ghd8*/*DTH8*, *Ghd7*.*1* and *OsCOL4* have been identified in the latter pathway. These genes repress the transcription of *Hd3a* and *RFT1* (*Rice Flowering Locus T 1*) by downregulating *Ehd1* expression independent of *Hd1* in LD^30^. The *OsGI*-*Hd1*-*Hd3a* and *OsGI*-*Ehd1*-*Hd3a* pathways have been demonstrated to function as independent pathways in controlling heading date at the early stage^20^. However, recent studies have shown that *Hd1* represses the expression of *Ehd1* in LD^33, 34^, suggesting that *Hd1*- and *Ehd1*-mediated pathways are not always independent. In a recent report, Heading Date Repressor 1 (HDR1) and OsK4 were found to suppress heading date by upregulating *Hd1* and downregulating *Ehd1*, and the HDR1-OsK4 interaction complex can phosphorylate Hd1^35^.

Zhang *et al*.^36^ showed that in a mixed Zhenshan 97 (ZS97) and Miyang 46 genetic background, the *Hd1* allele of ZS97 expresses photoperiod sensitivity and promotes heading in SD but delays heading in LD^36^. In contrast, in the ZS97 background, the *Hd1* allele of Miyang46 exhibits photoperiod insensitivity, delayed flowering and increased plant height and grain productivity under either LD or SD conditions^36^. Therefore, the dual functions of *Hd1* are dependent on the genetic background. However, it is not clear which gene in the genome background coordinates the photoperiod sensitivity of *Hd1*. In this study, 2 sets of Hd1 near isogenic lines in ZS97 and Minghui 63 (MH63) backgrounds exhibiting distinct photoperiod patterns are used as basic materials. Our objectives are (1) to accurately characterize the photoperiod of Hd1; (2) to identify which gene is interacted with *Hd1*; and (3) to elucidate how the function of Hd1 reverses between LD and SD.

## Results

### Validation of *Ghd6*

**Figure 1:**
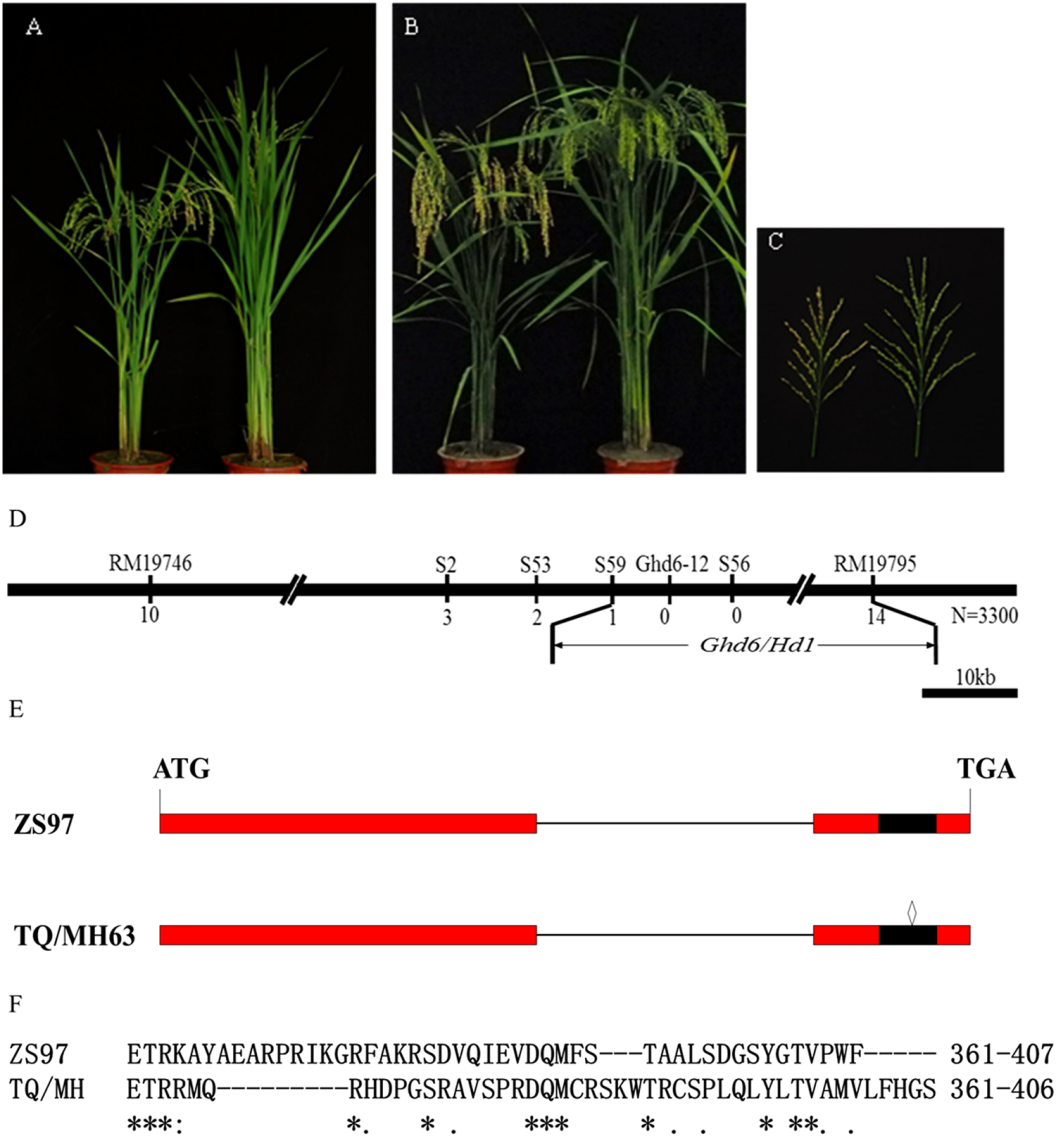
Performance of near isogenic lines of *Ghd6*/*Hd1* and map-based cloning of *Ghd6*/*Hd1*. (**A**) Phenotype of the whole plants for NIL-ZS (left) and NIL-TQ (right), (**A**) the picture was taken when NIL-TQ was flowering; (**B**) the picture was taken when NIL-ZS was ripening; (**C**) main panicles for NIL-ZS (left) and NIL-TQ (right); (**D**) Map-based Cloning of *Hd1*; (**E**) comparison sequences of *Hd1* of Teqing/Minghui 63 allele and Zhenshan 97 allele. The red rectangle represents the coding region of *Hd1*. The black region means the CCT domain of *Hd1* from +1012 bp to +1146 bp. The black line means the intron. Diamond indicates the position of a 4-bp (AAAG) deletion in CCT domain on +1089 site. (**F**) The frame shift mutation in CCT domain in Teqing and Minghui 63 allele.

A BC_4_F_1_ plant with ZS97 as the recurrent parent and Teqing (TQ) as the donor parent headed later than ZS97. Genotyping of 150 SSR markers evenly distributed throughout the whole genome showed that the plant carried a heterozygous region between RM19746 and RM19795 on chromosome 6, and no other heterozygous regions were checked. A BC_4_F_2_ population of 219 plants were developed by selfing the BC_4_F_1_ plant. The progeny test of the BC_4_F_2_ population confirmed the co-segregation for spikelets per panicle, heading date and plant height under natural long-day conditions (NLD) (Supplementary Figure 1). The segregation ratio fitted a 3:1 for a single dominance gene (χ^2^ = 0.95 < χ^2^_0.05_ = 3.84), indicating a pleiotropic gene between RM19746 and RM19795 controlling these trait variations. Subsequently, the gene was designated grains per panicle, plant height and heading date 6 (*Ghd6)*. QTL analysis showed that *Ghd6* explained approximately 78.5% of the observed heading date variance, with an additive effect of 6.0 d; 81.2% of plant height variance, with an additive effect of 7.7 cm; and 50.9% of the variance in spikelets per panicle, with an additive effect of 22.1 in the BC_4_F_2_ population (Supplementary Table 1). *Ghd6* exhibited partial dominance (Supplementary Table 1). Homozygous plants containing the ZS97 region of *Ghd6* (NIL-ZS) headed earlier, showing a shorter culm and smaller panicles than the TQ homozygotes (NIL-TQ) (Fig. 1A–C).

### Fine mapping and cloning of *Ghd6*

A total of 3300 BC_4_F_2_ individuals were assessed for heading date, plant height and spikelets per panicle. A total of 576 late-heading plants with a tall culm were selected for *Ghd6* fine mapping. First we screen the recombinants between Ghd6 and the flanking makers RM19746 and RM19795. In total, 10 and 14 recombinants were selected using the markers RM19746 and RM19795, respectively (Fig. 1D). Chromosome walking showed that *Ghd6* co-segregated with markers of Ghd6-12 and S56, indicating that *Ghd6* might localize to the region flanked by these two markers. The gene *Hd1*/*Loc_Os06g16370* encoding a zinc finger transcription factor located in the target region has been reported to control heading date. Probably it was the candidate gene of *Ghd6*. Hence, we sequenced the *Hd1* alleles of ZS97, TQ, NIL-ZS and NIL-TQ. ZS97 and NIL-ZS shared the same *Hd1* sequence, which differed from that shared by TQ and NIL-TQ. Compared with the ZS97 allele, the TQ allele harbored a 4-bp (AAAG) deletion in the CCT domain at the +1089 bp site, leading to a frame-shift mutation resulting in loss of *Hd1* function (Fig. 1E,F). All these 576 late heading plants exhibited the same genotype as TQ with the 4-bp deletion. Therefore, *Hd1* was the most likely candidate gene for *Ghd6*.

### Complementation test of *Hd1*

**Figure 2:**
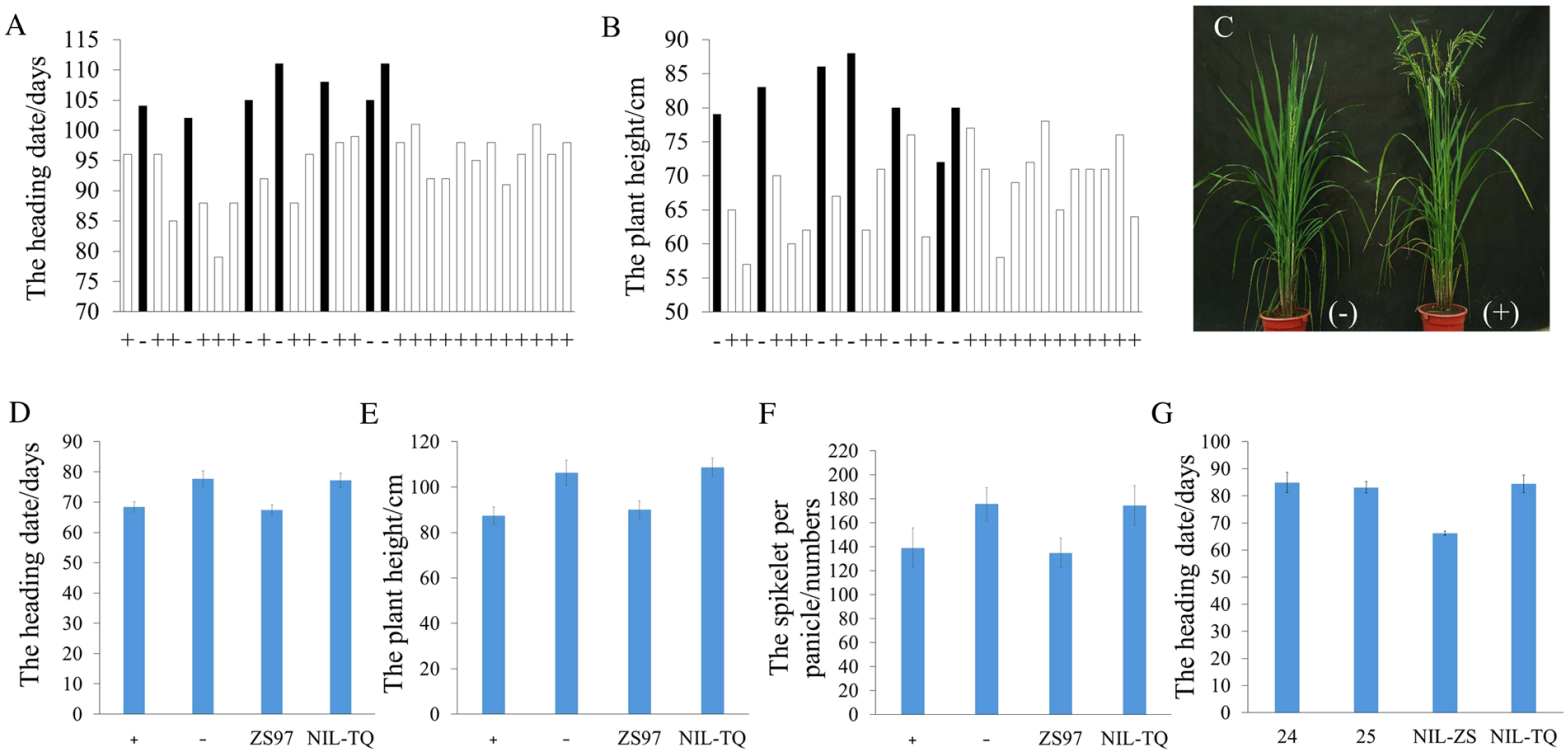
Phenotypes of *Hd1* transformation plants. (**A**) The heading date of T1 individuals from T0 plant 85 by transforming *ProHd1:Hd1:GFP* into NIL-TQ; (**B**) The plant height of T1 individuals from T0 plant 85; (**C**) a picture for homozygous positive and negatives T2 plants from T0 plant 85; (**D**) the heading date of T2 plants by selfing 85; (**E**) the plant height of T2 plants by selfing 85; (**F**) the spikelet per panicle of T2 plants by selfing 85; (**G**) the heading date of *Hd1* knock out plants 24 and 25 in T1 generation. “+” and “−” means the positive and negative transformed plants, respectively.

To determine whether *Hd1* was *Ghd6*, the 1450-bp fragment upstream of the translation start codon (ATG) and the complete coding region of *Hd1* from ZS97 were introduced into the PFA1300-GFP vector and transformed into NIL-TQ. In the T1 family of T0 plant 85, the positive transformed plants exhibited a significantly earlier heading date than the negative plants in Hainan (natural short-day conditions, NSD) (Fig. 2A). All but unhealthy one of the 7 negative plants displayed a taller plant height (Fig. 2B). One positive homozygous line and one negative line from T2 generation of T0 plant 85 were chosen to recheck the phenotype variation, the positive homozygous lines still exhibited a significantly earlier heading date, shorter plant height and fewer spikelets per panicle than the negative lines under NLD (Fig. 2C–F). Generations T1 and T2 from T0 plant 86 displayed a similar phenotype in NSD and NLD (Supplementary Table 2). In addition, several *Hd1* knockout mutants in NIL-ZS were created using the CRISPR-Cas9 method. As expected, knockout mutants 24 and 25, harboring different mutations, headed later than NIL-ZS by 18.7 and 16.9 days, respectively. Their heading dates were similar to NIL-TQ, which flowered later than NIL-ZS by 18.2 days (Fig. 2G). Taken together, the results indicated that *Hd1* was the pleiotropic gene underlying *Ghd6*.

### Response of *Ghd6* to photoperiod

When NIL-ZS and NIL-TQ plants were treated under LD and SD (8 or 10 hours of natural light, followed by 16 or 14 hours of dark), they generally did not react to the photoperiod, as neither genotype exhibited a significant difference in heading between SD and LD. Moreover, NIL-ZS consistently headed approximately 13–15 days earlier than NIL-TQ in 2013, regardless the day-length conditions under which the plants were grown, and 15–17 days earlier in 2014 (Table 1). In addition, the differences in the single plant yield and spikelets per panicle remained stable (40% approximately increasing in NIL-TQ) across LD and SD (Table 1). The results indicated that *Hd1*/*Ghd6* was insensitive to the photoperiod, which was not consistent with a previous report on *Hd1* ^19^.

**Table 1:** Performance of near isogenic lines for *Ghd6*/*Hd1* under short day and long day conditions.

Traits	Genotype	Day length (2013)			Day length (2014)	
		8 hrs	10 hrs	>13.5 hrs	10 hrs	>13.5 hrs
HD	NIL-ZS	65.4 ± 1.1**	67.7 ± 2.0**	67.3 ± 2.0**	63.6 ± 1.5**	65.7 ± 1.9**
	NIL-TQ	79.1 ± 2.1	81.6 ± 2.6	80.0 ± 1.4	78.5 ± 2.1	82.4 ± 2.1
PH	NIL-ZS	73.3 ± 2.1**	77.9 ± 3.8**	84.6 ± 1.3**	77.2 ± 2.2**	83.2 ± 4.1**
	NIL-TQ	88 ± 1.8	88.6 ± 2.6	101.6 ± 2.8	95.7 ± 2.8	101.7 ± 2.9
SPP	NIL-ZS	130 ± 4.1**	126.7 ± 5.5**	131.4 ± 3.7**	117.0 ± 8.5**	122.4 ± 10.1**
	NIL-TQ	182.8 ± 10.6	179.8 ± 10.9	171.8 ± 7.0	170.5 ± 10.3	175.5 ± 7.4
YD	NIL-ZS	17.8 ± 2.3**	17.8 ± 2.2**	18.2 ± 1.8**	16.7 ± 2.6**	17.7 ± 2.0**
	NIL-TQ	24.6 ± 1.9	25.1 ± 2.8	24.8 ± 2.2	24.1 ± 2.5	24.8 ± 2.5

HD, heading date; PH, plant height; SPP, spikelets per panicle; YD, yield; >13.5 hrs the natural day length is more than 13.5 hours in the period from sowing to heading (May 10 to August 5) in Wuhan; NIL-ZS, Homozygotes plants containing ZS97 region of *Ghd6*/*Hd1* in ZS97 background; NIL-TQ, Homozygotes plants containing TQ region of *Ghd6*/*Hd1* in ZS97 background. “**” mean the significantly difference by Duncan test P ≤ 0.01 when NIL-ZS compared to NIL-TQ.

### *Hd1* upregulates *Ehd1* expression in ZS97 background, regardless of day length

**Figure 3:**
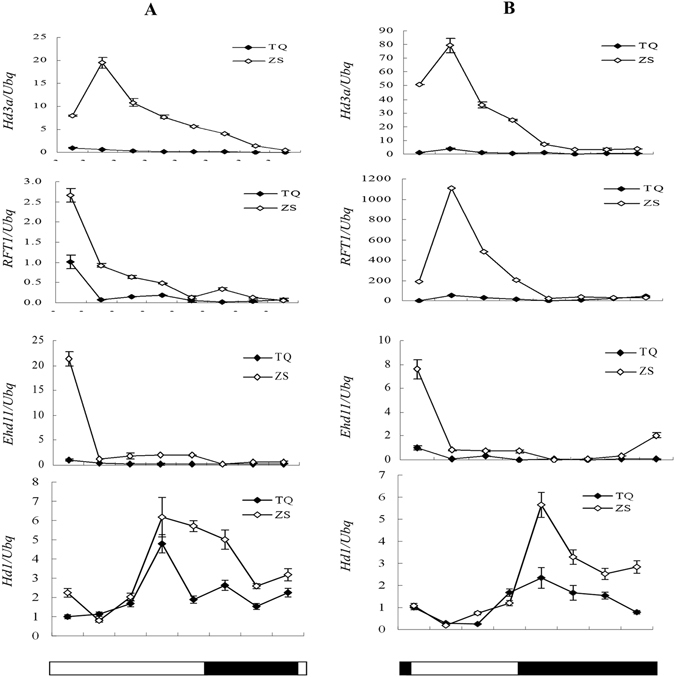
The expression of key heading date genes in NIL-ZS and NIL-TQ under LD and SD conditions. (**A**,**B**) The expression of *Hd1*, *Ehd1*, *Hd3a*, and *RFT1* in NIL-ZS and NIL-TQ under LD and SD.

In ZS97 background, *Hd1* promoted heading date in both LD and SD. To address this phenomenon, the expression of *Hd1*, *Ehd1*, *Hd3a* and *RFT1* in NIL-ZS and NIL-TQ was investigated under LD and SD. The expression levels of *Ehd1*, *Hd3a* and *RFT1* in NIL-ZS were significantly higher than that in NIL-TQ under both LD and SD, indicating that *Hd1* promoted the expression of *Hd3a* and *RFT1* through upregulation of *Ehd1* (Fig. 3A,B), resulting in early flowering, regardless of day length.

### Photoperiod sensitivity of *Hd1* in MH63 background

A previous study showed that *Hd1* delayed heading date in LD but promoted heading date in SD. These inconsistent results indicated that genes hidden in the genome control alternative photoperiod sensitivity. To identify the gene(s) involved, two reciprocal *Hd1* introgression lines, ZS-hd1 and MH-Hd1, from a cross between ZS97 and MH63 were screened. ZS-hd1 carried a non-functional MH63 *hd1* allele in ZS97 background, which shared the same coding region as the *hd1* allele, similar to TQ (Fig. 1E,F). MH-Hd1 carried a functional *Hd1* from ZS97 in MH63 background. To understand the genetic background of ZS-hd1 and MH-Hd1, the RICE6K SNP array was employed for the analysis of both genotypes (Supplementary Figure 2). Few regions segregated in both genotypes. However, no QTLs other than *Hd1* were located in the introgression segments, indicating that *Hd1* was the unique factor responsible for the altered heading dates. Again, ZS97 harboring functional *Hd1* headed earlier than ZS-hd1 in both NLD and NSD, by 11.3 and 17.4 days, respectively (Supplementary Figure 3A), consistent with the situation observed between NIL-ZS and NIL-TQ in LD and SD. However, compared with MH63, MH-Hd1 showed clear photoperiod sensitivity, with heading being delayed under NLD by 13.3 days and promoted under NSD by 23.7 days (Supplementary Figure 3A). Expression analysis showed that in LD, *Hd1* upregulated *Ghd7* and downregulated *Ehd1*, *Hd3a* and *RFT1* in the morning (8:30 am), when these genes should be highly expressed (Supplementary Figure 3B). This expression pattern is consistent with the delayed heading performance observed in LD. In SD, *Hd1* upregulated *Ehd1*, *Hd3a* and *RFT1* in the morning (8:30 am) and at midnight (0:30 am) (Supplementary Figure 3C) and led to MH-Hd1 heading earlier than MH63. Hence, the photoperiod sensitivity of *Hd1* was dependent on the genetic background.

### Genetic interaction between *Hd1* and *Ghd7*

**Figure 4:**
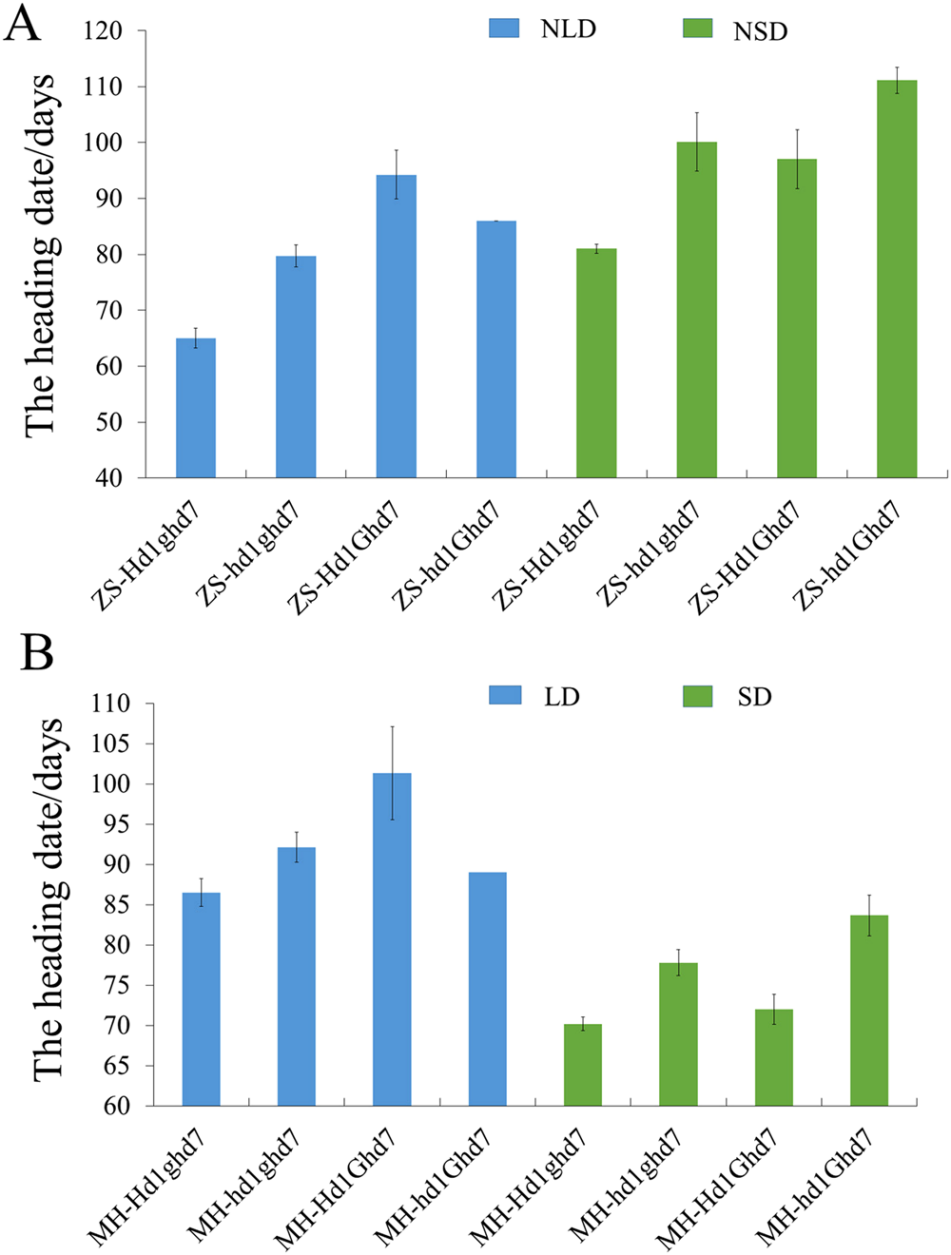
Genetic interaction on heading date between *Ghd7* and *Hd1* under long day and short day conditions. The heading date of 4 homozygous combinations between *Ghd7* and *Hd1* in Zhenshan 97 background (**A**) and Minghui 63 background (**B**). “NLD”, nature long days in Wuhan, Hubei; “NSD”, nature short days in Lingshui, Hainan. “LD”, controlled long day-length of 14 hours light; “SD”, controlled short day-length of 8 hours light.

The genetic interaction between *Hd1*, *Ghd8* and *Ghd7* has been reported to greatly delay flowering^34^. ZS97 and MH63 do not carry a functional *Ghd8* gene, while ZS97 harbors a functional *Hd1* and a deletion of *Ghd7*, and MH63 possesses *Ghd7* and a non-functional *hd1* ^16, 18, 34^. We hypothesized that *Ghd7* is the background determinant affecting the photoperiod sensitivity of *Hd1*. To confirm this hypothesis, we developed two F_2_ populations by crossing ZS-hd1 with ZS-Ghd7 carrying a functional *Ghd7* from MH63 and crossing MH-Hd1 with MH-ghd7 possessing a non-functional *ghd7* from ZS97. In NLD, *Ghd7* significantly interacted with *Hd1* in determining the heading date in ZS97 background (Table 2). Under LD, *Hd1* promoted heading date by 14.7 and 5.6 days without *Ghd7* in ZS97 and MH63 backgrounds, respectively, and *Hd1* correspondingly repressed heading date by 8.3 and 12.3 days in the presence of *Ghd7* (Fig. 4A,B). Accordingly, the effect of *Ghd7* on delaying heading date was significantly altered between the *Hd1* and *hd1* backgrounds. *Ghd7* in the ZS97 background with *Hd1* delayed heading by 29.3 days, but the delay was decreased to 6.3 days without *Hd1*, while *Ghd7* in MH63 background with *Hd1* delayed heading by 14.8 days, but promoted heading by 2–3 days without *Hd1* (Fig. 4A,B). In SD, regardless of the presence or absence of *Ghd7*, *Hd1* consistently promoted the heading date by 14.1 and 19 days in the ZS97 background and by 11.6 and 7.4 days in the MH63 background (Fig. 4A,B). In the ZS97 background, *Ghd7* repressed heading date by 16 days with *Hd1* or 11 days without *Hd1* (Fig. 4A,B). In MH63 background, the effect of *Ghd7* in repressing heading date sharply decreased, regardless of *Hd1* (Fig. 4A,B).

**Table 2:** Two-way ANOVA in the F2 population from the cross between ZS-hd1 and ZS-Ghd7.

Source of genetic variation	F	*P*	SSE in SST%^a^
General			
*Hd1*	6.2	0.0046	3.3
*Ghd7*	47.9	3.2E-11**	25.9
*Hd1 xGhd7*	20.8	3.1E-9**	22.4
*Partition*			
*AA*	29.7	3.0E-6**	
*AD*	12.5	0.0001	
*DA*	3.0	0.09	
*DD*	1.1E-3	0.97	

^a^Percent of effect sum of squares in the total sum of squares.

### Expression analysis of genes involved in the photoperiod pathway

After detecting the genetic interaction between *Hd1* and *Ghd7*, we examined whether the expression patterns of key flowering genes also exhibited subtle changes in the presence of different gene combinations. Hence, gene expression was assessed in four homozygotes for *Hd1* and *Ghd7* in ZS97 background. In SD, no transcriptional regulation was observed between *Ghd7* and *Hd1* (Supplementary Figure 4). However, *Hd1* upregulated *Ghd7* in LD (Supplementary Figures 3B and 4). In LD, *Ghd7Hd1* showed strongly suppressed expression of *Ehd1* and *Hd3a* than *Ghd7hd1* and *Ghd7Hd1*, resulting in the latest heading. In SD, *Ghd7Hd1* exhibited moderately suppressed expression of *Ehd1* and *Hd3a*, resulting in the second earliest heading among these 4 genotypes. The expression patterns of florigen genes were obviously dependent on the combination of *Ghd7* and *Hd1* genes. *RFT1* is also an important florigen gene in the heading date pathway; however, a previous study showed that *RFT1* is a nonfunctional allele in ZS97 background^37^. Therefore, we did not examine the expression of the *RFT1* in ZS97 background, and a yeast one-hybrid assay showed that both Ghd7 and Hd1 could not bind to the promoter of *Ehd1* from −1341 bp to +47 bp (Supplementary Figure 5).

### Physical interaction between Ghd7 and Hd1

**Figure 5:**
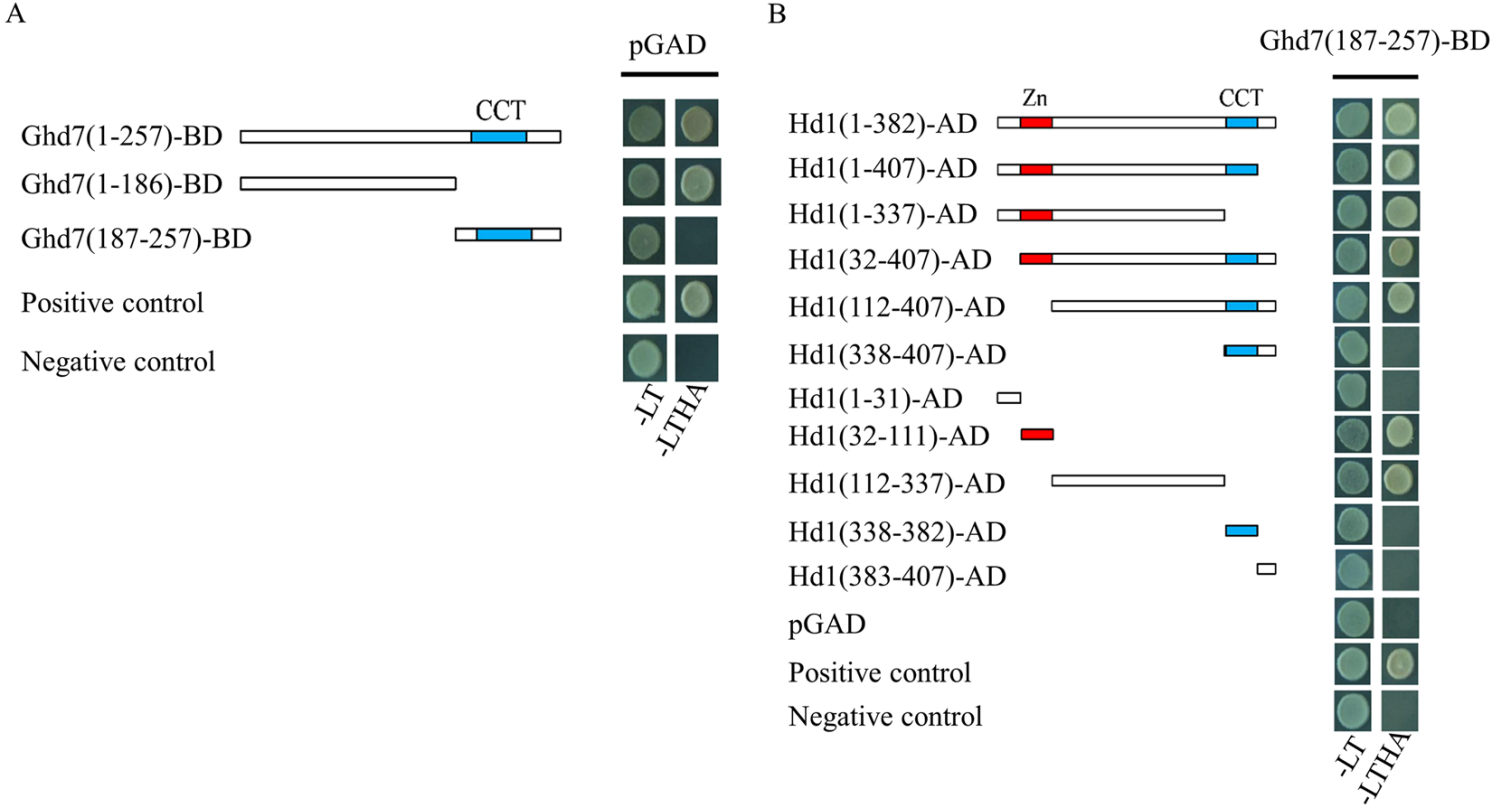
The physical interaction between Ghd7 and Hd1. (**A**) Selfing activation of Ghd7 in Yeast; (**B**) interaction between Ghd7 and Hd1 in Yeast. “-LT” means the medium lack of Leu and Trp. “-LTHA” means medium lack of Leu, Trp, His and Ade.

*Ghd7* has been reported to repress heading via transcriptional regulation of *Ehd1*, but not of *Hd1* ^16^. It was considered most likely for Ghd7 to interact with Hd1 at the protein level. To confirm this potential interaction, the yeast two-hybrid system was used. Analysis of the transcriptional activation of Ghd7 revealed that Ghd7 presented the ability to undergo self-activation, conferred by the segment from amino acids 1 to 186 (Fig. 5A). Subsequently, the segment of Ghd7 (aa187–257) containing the CCT domain was used to test hybridization with Hd1 in a yeast two-hybrid assay. Significant interactions were observed between the Ghd7 (aa187–257) and Hd1 (aa32–111) segments harboring the zinc finger domain and the Hd1 segment (aa112–337) between the zinc finger domain and the CCT domain (Fig. 5B).

### Ghd7 inhibits the transcriptional activation activity of Hd1

In CONSTANS (CO), the ortholog of Hd1 in Arabidopsis, transcriptional activation is conferred by the region between the zinc finger and CCT domains^38^. Thus, we suggested that Hd1 acts as a transcription activator and that the region (aa112–337) between the zinc finger and CCT domain confers this activity. Subsequently, several variants of Hd1 were cloned in-frame into the effector vector containing the GAL4 DNA-binding domain of yeast and co-transformed with a reporter vector into rice protoplasts for transcriptional activation activity assays (Supplementary Figure 6A). The relative LUC activity activated by GAL4-Hd1^ZS^, GAL4-Hd1^NIP^, GAL4-Hd1^TQ^, GAL4-Hd1^ZS337^ and GAL4-Hd1 ^ZS111^ was 117.2-, 50.4-, 2.1-, 828.5- and 1.8-fold higher than in the GAL4-YFP control, respectively (Supplementary Figure 6B). These results suggested that Hd1 possessed transcriptional activation conferred by the region (aa112–337) between the zinc finger and CCT domains. The transcriptional activation activity of the TQ allele of Hd1 was 4.2 percent that of the ZS97 allele(Supplementary Figure 6B), indicating that the mutation in the CCT domain of Hd1 in the TQ allele might destroy its transcriptional activation activity, leading NIL-TQ to undergo late flowering compared with NIL-ZS flowering.

Accordingly, to survey whether Ghd7 affected the transcriptional activation of Hd1, the 35 S:Ghd7-CFP and GAL4-Hd1^ZS^ vectors were co-transformed at different dosages into rice protoplasts, and analysis was performed via transcriptional activation assays. The relative LUC activity activated by GAL4-Hd1^ZS^ gradually decreased from 7.0 to 0.8 with an increasing dosage of Ghd7-CFP (Supplementary Figure 6B), indicating that Ghd7 repressed the transcriptional activation activity of Hd1.

## Discussion

### *Hd1* essentially acts as a promoter of the heading date in rice

In the present study, the heading date gene *Ghd6* in ZS97 was found to be insensitive to photoperiod, promoting heading regardless of day length. Gene cloning showed that *Hd1* was the gene underlying *Ghd6*. Thus *Hd1* is photoperiod insensitivity in ZS97, which was not consistent with a previous report that *Hd1* promotes heading in SD but delays heading in LD^19^. Photoperiod insensitivity of *Hd1* was also previously observed in *Hd1* mutant that delayed heading regardless of day length as compared to its wild type^39^. Moreover, the *Hd1* allele from ZS97 promotes heading in both LD and SD^36^. Here, we further confirmed that *Hd1* upregulated *Ehd1*, *Hd3a* and *RFT1* in LD and SD and acted as a transcriptional activator; these findings are identical to the most recent study in which Hd1 was shown to activate the expression of Hd3a^40^. Thus, Hd1 essentially acts as a promoter of heading date in rice without *Ghd7* (Supplementary Figure 6B).

### The alternative functions of *Hd1* are dependent on *Ghd7* in LD

It is clear that *Hd1* possesses opposite functions in the regulation of heading date under different day-length conditions^19^. Additional surveys have consistently confirmed the bifunctional character of *Hd1* ^20, 36, 39, 41^. In this study, we found that *Hd1* essentially acts as a promoter of heading date in rice, but it delays heading in some genetic backgrounds such as the MH63 background in LD. These indicate function of *Hd1* is genetic background dependent. The heading date performance of two sets of *Hd1* NILs in the present study indicated that *Ghd7* is the determinant of *Hd1* bi-functionality. Without *Ghd7*, *Hd1* promotes heading date in LD and SD by upregulating its downstream genes, including *Ehd1*, *Hd3a* and *RFT1*. Under LD, proteins of Ghd7 and Hd1 assemble into a complex through binding of the Ghd7 CCT domain to the transcription activation domain of Hd1. It has been suggested that the interaction between Ghd7 and Hd1 might block or weaken the transcriptional activation activity of Hd1 and release the transcriptional repression activity of Ghd7, thereby repressing the expression of downstream genes of *Ehd1*, *Hd3a* and *RFT1*, consequently delaying heading date in LD. It has been reported that *Ghd7* expression is significantly lower in SD than in LD^16^. Thus, we hypothesized that this situation likely impairs or weakens the interaction between Ghd7 and Hd1 in SD. Therefore *Hd1* still promotes the heading date in a *Ghd7* background under SD. More evidence from biochemistry and structural biology analyses is needed to confirm this hypothesis.

In the presence of a functional allele of *Hd1*, *Ghd7* exerts a large effect on heading date. Without *Hd1*, *Ghd7* in ZS97 background has a much smaller effect on delaying heading, and the effect of *Ghd7* is almost absent in MH63 background (Fig. 4A,B). It is likely for this reason that a few varieties with strong *Ghd7* alleles have been observed in northeast China and Japan^34, 42^. Additionally, when *Ghd7* segregating population in ZS97 background was used for gene cloning, its effects were enhanced that made *Ghd7* cloning work feasible and successful^16^. Taken together, these findings indicate that the photoperiod sensitivities of *Ghd7* and *Hd1* are dependent on each other.

### Integration of Hd1-mediated and Ehd1-mediated photoperiod pathways

**Figure 6:**
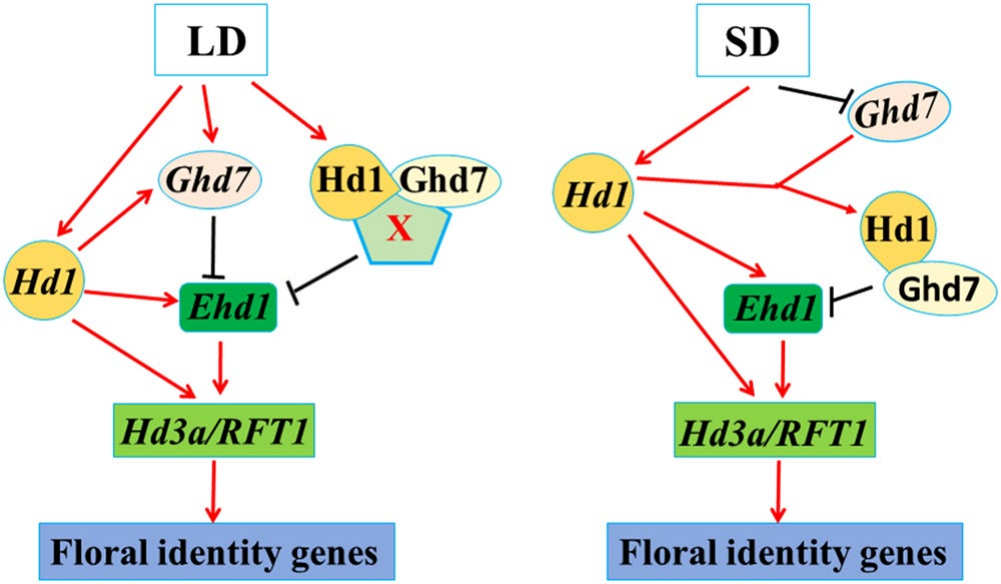
A model for the regulation of *Ehd1* by *Ghd7* and *Hd1*. *Hd1* alone promoted the expression of *Ehd1* in LD and SD; *Ghd7*, without *Hd1*, repressed *Ehd1* in LD; The interaction between *Ghd7* and *Hd1* was occurred, and repressed the expression of *Ehd1* together with an unknown factor X in LD.

Rice exhibits two photoperiod pathways that regulate heading, mediated by *Hd1* and *Ehd1*. Previous studies suggested that these two pathways are independent^20^. Recent studies have indicated the *Ehd1*-mediated pathway is dominant, while *Hd1*-mediated pathway likely functions via the *Ehd1* pathway^40^. The *Ghd7Hd1* genotype displays significantly delayed heading under LD. At the transcriptional level, *Ghd7Hd1* significantly downregulates *Ehd1* in LD. Moreover, interactions were detected between Ghd7 and Hd1. However, these proteins do not directly bind to the promoter of *Ehd1*. ChIP analysis showed that Ghd7 was enriched at the promoter of *Ehd1*, but there were no cis-elements in the promoter of *Ehd1* ^40^. It is likely that Ghd7 and Hd1 are involved in a repression complex containing components that directly bind to the promoter of *Ehd1*. Ghd7 acts as a repressor of *Ehd1*, independent of *Hd1* ^40^, whereas *Hd1* is an activator of *Ehd1*. When these proteins interacted under LD, the repression function of *Ghd7* was observed, while under SD, the promotion function of *Hd1* was primarily recorded. Considering these findings together, we suggest the existence of a regulatory network involving Ghd7 and Hd1 (Fig. 6). In SD, the interaction between Ghd7 and Hd1 was not observed. *Hd1* promotes *Hd3a* expression either directly or via the upregulation of *Ehd1*, resulting in early heading. Under LD, without *Ghd7*, *Hd1* alone upregulated *Ehd1* and further upregulated *Hd3a*, ultimately leading to early heading. Without *Hd1*, *Ghd7* alone suppressed *Ehd1* and *Hd3a*, resulting in late heading. In the presence of *Ghd7*, *Hd1* upregulated *Ghd7*, consistent with a previous report^33^, and a repressor protein complex including Ghd7, Hd1 and components of unknown identity was formed and suppressed *Ehd1* and *Hd3a*/*RFT1*, resulting in markedly late heading.

In summary, Hd1 promotes heading without Ghd7 regardless of day length but represses heading through interactions with Ghd7 under LD. The interaction between Ghd7 and Hd1 determines their corresponding photoperiod sensitivities. The involvement of the transcription-activating domain of Hd1 in the protein-protein interaction likely abolishes its transcription-activating activity. The interaction between Ghd7 and Hd1 integrates the Hd1-mediated and Ehd1-mediated photoperiod flowering pathways. These findings provide new insight into the function of *Hd1* and *Ghd7* in the photoperiodic flowering pathway in rice.

## Materials and Methods

### Plant materials

A BC_4_F_2_ population showing varied heading dates was developed using ZS97 as the recurrent parent and Teqing (TQ) as the donor parent. The BC_4_F_2_ plants exhibiting homozygous TQ and ZS97 introgression fragments containing *Ghd6*/*Hd1* were designated NIL-TQ and NIL-ZS, respectively. A total of 3300 BC_4_F_2_ plants were used to identify the gene underlying the variation of heading date. Introgression lines of *Hd1* in the ZS97 background and MH63 background were scanned from reciprocal advanced chromosome segment substitution lines (CSSLs) between ZS97 and MH63^6^] and will hereafter be referred to as ZS-hd1 (MH63 homozygous *Hd1* alleles in ZS97 background) and MH-Hd1 (ZS97 homozygous *Hd1* alleles in MH63 background), respectively. ZS-Ghd7, a NIL carrying MH63 *Ghd7* alleles in ZS97 background^16^, was crossed with ZS-hd1, and a near isogenic F_2_ population was obtained to analyze the genetic interaction between *Hd1* and *Ghd7*. A NIL without *Ghd7* in MH63 background was also selected from the CSSLs, which was designated MH-ghd7.

### Field experiments and photoperiod treatment

The plant materials were sown in the middle of May, and 25-day-old seedlings were transplanted to the field at the same planting density, with a distance of 16.5 cm between plants within a row and 26.5 cm between rows. The plants were subsequently grown under natural long-day field conditions (NLD), in which the day length was more than 13.5 hours, from the middle of May to the beginning of August at the experimental station of Huazhong Agriculture University, Wuhan, China (31°N latitude). For the experiments involving natural short-day field conditions, the experimental materials were sown in Lingshui, Hainan (18°N latitude) at the beginning of December and were transplanted to the field after 1 month, at the same planting density as in Wuhan, and grown under an average day-length of less than 12 hours from December to the middle of March. For the photoperiod treatment, the seedlings were initially grown in the natural field for 20 days under natural long days. Subsequently, 5 plants of each genotype were transplanted in parallel to the fields under NLD (LD) and short-day conditions (SD), with a day length of 8 or 10 hours and darkness of 16 or 14 hours in the field, and were covered with a black cloth until the growth phase transition of late heading plants was initiated.

### Trait measurement and data analysis

HD was individually measured as the days from sowing to the emergence of the first panicle in the plant. The number of spikelets per panicle (SPP) was recorded as the total number of spikelets divided by the number of panicles. The grain yield (YD) was calculated as the grain weight per plant. Plant height (PH) was measured from the surface to the top of the main panicle. For the fine mapping of *Ghd6* in the BC_4_F_2_ population, interval mapping was employed to identify the QTL underlying heading date and plant height using MAPMAKER/EXP and MAPMER/QTL^43^. The statistical significance of the two-way genetic interaction was assessed via an orthogonal contrast test using the program STATISTICA 8.0. Clustal Omega 1.2.2 was employed for comparisons of the Hd1 protein sequence (http://www.clustal.org/omega/).

### Transformation of *Hd1*

To construct the *ProHd1:Hd1:GFP* vector, a 1450-bp fragment upstream of the coding start site and the CDS without the terminator of *Hd1* from ZS97 were cloned into the PFA1300-GFP vector using the primers ProHd1-1450 and Hd1-GFP (Supplementary Table 3). PFA1300-GFP is a remolded PCAMBIA1300 vector fused with the *GFP* sequence as a tag. The *ProHd1:Hd1:GFP* plasmid was subsequently transformed into NIL-TQ by means of *Agrobacterium*-mediated transformation^44^.

To obtain the *hd1* mutant, we used the vector pCXUN-Cas9 to knock out functional *Hd1* in NIL-ZS lines. The target sequence was obtained from the website http://cbi.hzau.edu.cn/cgi-bin/CRISPR ^45^. The target sequence started with an “A” base, since we used the OsU3 promoter (Supplementary Table 3). Subsequently, we employed the overlapping PCR method to obtain gRNA expression cassettes^46^. The pOsU3-gRNA plasmid was used as a template for two rounds of PCR. The first round of PCR was performed using the primers *Hd1*-CRP-F and OsU3-R, and the second was performed used the primers *Hd1*-CRP-R and OsU3-F (Supplementary Table 3). We mixed the two obtained products for subsequent extraction and purification. Finally, the mixed product was employed for a third round of PCR, which was conducted using the primers OsU3-R and OsU3-F. Daniel Gibson’s enzymatic assembly method was employed to clone the gRNA fragment into the pCXUN-Cas9 vector, which was linearized using FastDigest KpnI (Thermo Scientific, USA)^47^. Finally, the plasmids were introduced into *Agrobacterium* strain EHA105 by electroporation and subsequently transformed into NIL-ZS callus according to a previous study^44^. After the *Hd1* mutants were obtained, the primers *Hd1*-CRP-SeqF and *Hd1*-CRP-SeqR were used to examine the *Hd1* mutations (Supplementary Table 3).

### Analysis of diurnal gene expression

Seedlings were grown in pots under natural LD for 20 days and were subsequently transferred to an S10H growth chamber (Conviron, Canada), with half of the plants being grown under LD and the other half under SD. The growth conditions were set as follows: 14 h light and 10 h dark for LD, 10 h light and 14 h dark for SD; light intensity was set at 10,000 1x; and the temperature was 30 °C in the light period and 26 °C in the dark period. After treatment for 7 days, leaf samples for RNA extraction were collected from the LD and SD treatments at 3-h intervals over a 24-h period starting at 08:30. For each time point, leaves from three different plants were harvested as biological replicates. Total RNA was extracted using the TRIzol reagent (Invitrogen, San Diego, CA, USA) and treated with DNase I (Invitrogen). cDNA was synthesized from 3 μg of RNA using SuperScript III Reverse Transcriptase (Invitrogen). The quantitative analysis of gene expression was performed with SYBR Premix Ex Taq (TakaRa, Otsu, Japan) on an Applied Biosystems 7500 Real-time PCR System (Applied Biosystems, Foster City, CA, USA). The data were analyzed using the relative quantification method. Three biological replicates for every sample were prepared at every time point, and every sample was examined with three technical repeats. The mean data across the three technical replicates were regarded as the original data for one biological replicate, and the mean data across the three biological replicates were regarded as the final data for the comparative analysis. In the present study, four major heading date genes were analyzed during diurnal expression in LD and SD. The primers used for real-time PCR are listed in Table Supplement 3.

### Yeast one-hybrid assay

We followed the method described by Wang *et al*.^48^ to conduct the yeast one-hybrid assay^48^. The −1786 to +47 region of *Ehd1* from Nippobare was truncated into 4 fragments via PCR and inserted into the Placzi2μ vector (P1:LacZ, P2:LacZ, P3:LacZ, and P4:LacZ) (Supplement Table 3). The CDSs of *Ghd7* (MH63 allele) and *Hd1* (ZS97 allele) were cloned into the PJG4-5 vector (Ghd7-AD and Hd1-AD) using the primers Ghd7-Y1H and Hd1-Y1H (Supplement Table 3). Subsequently, Ghd7-AD, Hd1-AD and empty PJG4-5 were separately co-transfected with the five reporter vectors (the empty Placzi2μvector, P1:LacZ, P2:LacZ, P3:LacZ, and P4:LacZ) into the yeast strain EGY48. Empty PJG4-5 was also used as a control.

### Yeast two-hybrid assay for Ghd7 and Hd1

For the yeast two-hybrid assay, the variants of *Ghd7* and *Hd1* were cloned in-frame into the pGBKT7 (Ghd7-BDs) and pGADT7 (Hd1-ADs) vectors, respectively (Fig. 5). For the Ghd7 self-activation experiment, the Ghd7-BD plasmids were individually co-transformed with pGADT7 into yeast strain AH109. Subsequently, the cells were grown on -2 SD medium lacking Leu and Trp (-LT) and -4 SD medium lacking Leu, Trp, His and Ade (-LTHA). For the yeast two-hybrid analysis of *Ghd7* and *Hd1*, Ghd7-BD (187–257) was co-transformed with each of the Hd1-AD variants to AH109 to perform the assay of Ghd7 self-activation. All primers used are shown in Supplementary Table 3.

### Transcriptional activation activity analysis of Hd1

We followed a previously reported analysis method for assessing the transcriptional activation activity of Hd1^48^. The CDSs of *Hd1* were amplified from varieties ZS97 and Nipponbare with primers GAL4-Hd1-ZS. The CDS of TQ were amplified with primers GAL4-Hd1-TQ. Two truncated *Hd1* of ZS97 were amplified with GAL4-Hd1-337 and GAL4-Hd1-111. Then all *Hd1* fragments cloned into effect vectors fused with the GAL4-binding domain using primers (Supplementary Table 3; Supplementary Figure 6A). YFP was also cloned into effect vectors as a control check (CK). Subsequently, the effect vectors, the reporter vector carrying Luciferase and the internal control vector carrying GUS were co-transformed into protoplasts of NIL-TQ at a ratio 2:2:1 (μg). Simultaneously, the 35 S:Ghd7:CFP vector^18^, was co-transformed with the GAL4-Hd1effect vector (including the full-length CDS of Hd1 from ZS97), the reporter vector and control vector into the protoplasts of NIL-TQ at dosages of 2:2:2:1 (μg), 4:2:2:1 (μg) and 10:2:2:1 (μg), to investigate the influence of Ghd7 on the transcriptional activation activity of Hd1.

## Electronic supplementary material

Supplementary materials
