# Supplementary material for: Alternative functions of Hd1 in repressing or promoting heading are determined by Ghd7 status under long-day conditions
Source: Sci Rep. 2017 Jul 14;7:5388. doi: 10.1038/s41598-017-05873-1 (PMC5511259; doi:10.1038/s41598-017-05873-1)
Supplement: Supplementary file 1 — Supplementary materials [file 41598_2017_5873_MOESM1_ESM.doc]

**Alternative functions of Hd1 in repressing or promoting heading are determined by Ghd7 status under long-day conditions**

Zhanyi Zhang^#^, Wei Hu^#^, Guojing Shen, Haiyang Liu, Yong Hu, Xiangchun Zhou, Touming Liu, Yongzhong Xing*

**Supplementary materials**

Supplement Table 1. QTL effects estimated in the BC_4_F_2_ and BC_4_F_3_ population.

| Trait | Population | LOD | A^a^ | D^b^ | Var ^c^ (%) |
| --- | --- | --- | --- | --- | --- |
| SPP | BC_4_F_2_ | 30.0 | 22.1 | -9.8 | 50.9 |
|  | BC_4_F_3_ | 36.2 | 19.9 | -7.0 | 62.3 |
| GPP | BC_4_F_2_ | 14.1 | 11.6 | - | 31.4 |
|  | BC_4_F_3_ | 23.2 | 12.6 | -6.1 | 47.0 |
| YD (g) | BC_4_F_2_ | 4.1 | 2.0 | -1.1 | 9.9 |
|  | BC_4_F_3_ | 14.9 | 2.4 | -0.8 | 33.7 |
| PH (cm) | BC_4_F_2_ | 73.6 | 7.7 | -3.4 | 81.2 |
|  | BC_4_F_3_ | 51.0 | 5.9 | -1.5 | 74.2 |
| HD (day) | BC_4_F_2_ | 58.2 | 6.0 | -1.8 | 78.5 |
|  | BC_4_F_3_ | 60.2 | 6.1 | -1.9 | 80.1 |

Populations of BC_4_F_2_ and BC_4_F_3_ were grown under natural long day conditions in 2011 and 2012. A, additive effect, positive A means TQ allele increasing trait values. D, dominance effect, negative D means that the heterozygote values are smaller than the mean of parents. Var %, variance explained by QTL.

Supplement Table 2. The phenotype of the transgenic line 86 of *ProHd1:Hd1:GFP*

| Generation | Day-length | Traits | Positive plants | Negative plants | P-Value |
| --- | --- | --- | --- | --- | --- |
| T1 | NSD | HD (days) | 91.3±6.1 | 104.8±3.4 | <0.01 |
|  |  | PH (cm) | 67.7±4.5 | 79.8±1.7 | <0.01 |
| T2 | NLD | HD (days) | 68.6±1.6 | 76.6±4.1 | <0.01 |
|  |  | PH (cm) | 87±3.2 | 108.2±6.5 | <0.01 |
|  |  | SPP | 108.2±15.2 | 159.6±18.8 | <0.01 |

P-Value estimated by Duncan test.

Supplement Table 3 Primers used in this study

| Primer name | Forward primer 5'-3' | Reverse primer5'-3' |
| --- | --- | --- |
| RM19746 | ccacccatcctgtagatagtacg | ttacagaggagattaggagtgagg |
| RM19795 | tagtagttggcatctccggttgc | caagcggccactacgtatagtacc |
| S2 | acttgctggccaatgtatcc | gcccatgttcatataccttc |
| S53 | gaaggaggaggaggaggaga | caccaaaatacatgccgttg |
| S59 | gtcaggccttgttgaggaag | caccgattggtgggttttag |
| Ghd6-12 | tccatcatcctcctccctaa | acaaggacgagcttggagaa |
| S56 | gccaggaagtttgagaagac | ctgcacatctgatctcttgg |
| qRT-Hd1 | tcagcaacagcatatctttctcatca | tctggaatttggcatatctatcacc |
| qRT-Ehd1 | tggaaatctcgaaaaacccg | gcgctagcaaagcttcggt |
| qRT-Ghd7 | aggtgctacgagaagcaaatcc | gggcctcatctcggcatag |
| qRT-Hd3a | gctcactatcatcatccagcatg | ccttgctcagctatttaattgcataa |
| qRT-RFT1 | tgacctagattcaaagtctaatcctt | tgccggccatgtcaaattaataac |
| Ubq | gagcctctgttcgtcaagta | actcgatggtccattaaacc |
| Hd1-CRP | aacgtgttcgaccaggaggtgttttagagctagaaatagcaagtta | acctcctggtcgaacacgttgccacggatcatctgcacaac |
| OsU3 | cccctttcgccaggggtaccgtaattcatccaggtctccaag | acctcctggtcgaacacgttgccacggatcatctgcacaac |
| Hd1-CRP-Seq | tgcgaggtagaggaacaggaga | aaccactatgctgctgctcact |
| ProHd1-1450 | ctcggtaccgtcgaccaaccacagcataacaaactc | tctcgaggaacgaagtagccttgcttgtgg |
| Hd1-GFP | agagctcctcgagatgaattataattttggtggcaacg | tggatccgaaccatggaacagtaccatagc |
| P1-Y1H | gtacccggggatctgtcgacctcgagagctttgcacatatagctgc | tatacatacagagcacatgcctcgagatgaccagaactcttagtcc |
| P2-Y1H | gtacccggggatctgtcgacctcgagattacatgcatggaagaagg | tatacatacagagcacatgcctcgagtcgagtcactgatgatatgc |
| P3-Y1H | gtacccggggatctgtcgacctcgagattgcggttgtggaggaagg | tatacatacagagcacatgcctcgagtatgtagaaatagtagagg |
| P4-Y1H | gtacccggggatctgtcgacctcgagtccgtgtttgatccgattcc | tatacatacagagcacatgcctcgagagctcccttcctccacaacc |
| Ghd7-Y1H | tgccagattatgcctctcccgaattcatgtcgatgggaccagcag | caaagcttctcgagtcggccgaattcctatctgaaccattgtccaagc |
| Hd1-Y1H | tgccagattatgcctctcccgaattcatgaattataattttggtggcaacg | caaagcttctcgagtcggccgaattctcagaaccatggaacagtacc |
| Ghd7-Y2H | gaattcatgtcgatgggaccagca | ggatccgctatctgaaccattgtccaagct |
| Ghd7-Y2H(1-186) | gaattcatgtcgatgggaccagca | cgcggatcctgcgccaaccaccgtgtttggg |
| Ghd7-Y2H(187-257) | ccggaattcatggtggagagggaggccaagc | ggatccgctatctgaaccattgtccaagct |
| Hd1-Y2H-1 | ccggaattcatgaattataattttggtggcaac | cgcggatcctcagaaccatggaacagtaccatagc |
| Hd1-Y2H-2 | ccggaattcatgaattataattttggtggcaac | cgcggatccatctgatctcttggcgaaacg |
| Hd1-Y2H-3 | ccggaattcatgaattataattttggtggcaac | cgcggatccgtccatggagctgaagtgaagg |
| Hd1-Y2H-4 | ccggaattcgcgcggccgtgcgacgggtgc | cgcggatcctcagaaccatggaacagtaccatagc |
| Hd1-Y2H-5 | ccggaattcgccaggcgccaccagcgcgtc | cgcggatcctcagaaccatggaacagtaccatagc |
| Hd1-Y2H-6 | ccggaattcagggaggccagggtgctcag | cgcggatcctcagaaccatggaacagtaccatagc |
| Hd1-Y2H-7 | ccggaattcatgaattataattttggtggcaac | cgcggatccccatgggcagccgctcc |
| Hd1-Y2H-8 | ccggaattcgcgcggccgtgcgacgggtgc | cgcggatccgagcgggttcgcggagtgcacc |
| Hd1-Y2H-9 | ccggaattcgccaggcgccaccagcgcgtc | cgcggatccgtccatggagctgaagtgaagg |
| Hd1-Y2H-10 | ccggaattcagggaggccagggtgctcag | cgcggatccatctgatctcttggcgaaacg |
| GAL4-Hd1 | gcccgtcgacggatccggtaccatgaattataattttggtggcaacg | gggaattggatcttcaggtacctcagaaccatggaacagtaccatagc |
| GAL4-Hd1-TQ | gcccgtcgacggatccggtaccatgaattataattttggtggcaacg | gggaattggatcttcaggtacctcattcataacacagattgtctag |
| GAL4-Hd1-337 | gcccgtcgacggatccggtaccatgaattataattttggtggcaacg | gggaattggatcttcaggtacctcagtccatggagctgaagtgaag |
| GAL4-Hd1-111 | gcccgtcgacggatccggtaccatgaattataattttggtggcaacg | gggaattggatcttcaggtacctcagagcgggttcgcggagtgcac |


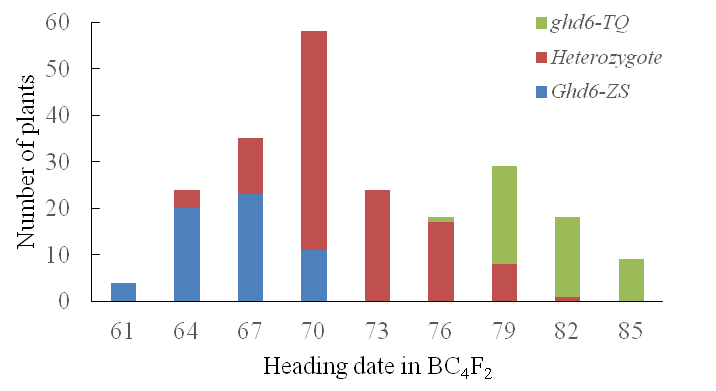


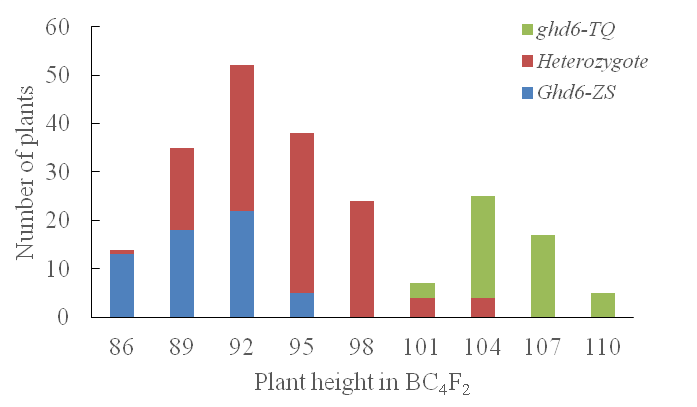


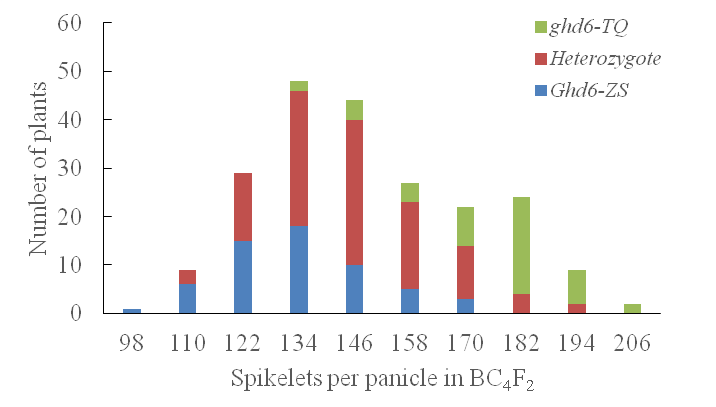


Supplementary Figure 1. Segregation in grain per panicle, heading date and plant height in BC_4_F_2_ generation under nature long-days


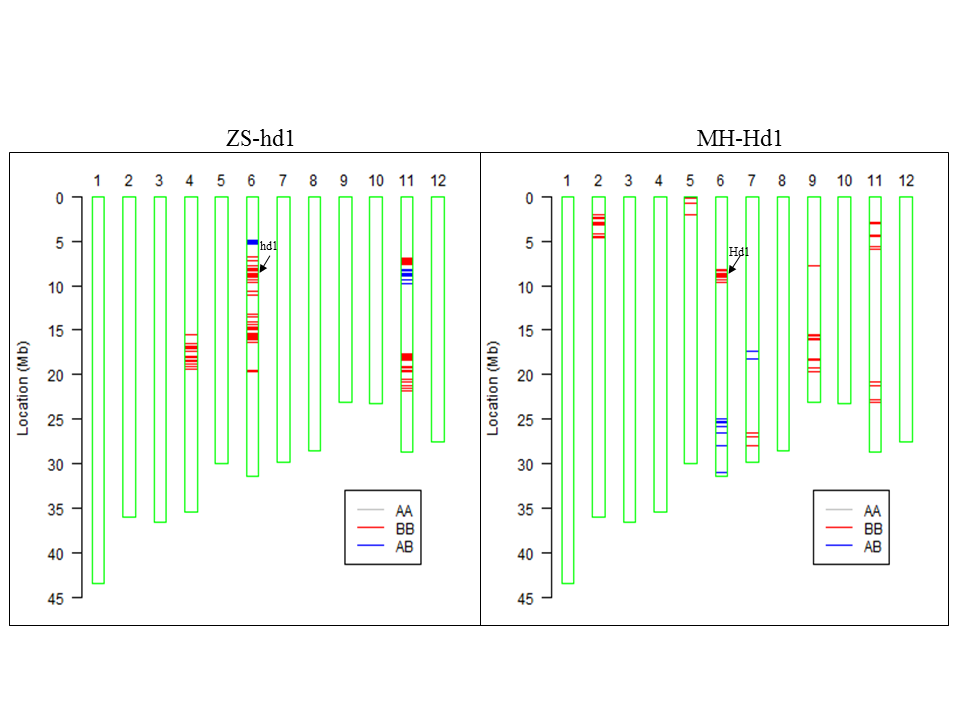


Supplementary Figure 2. Background scanning of the ZS-hd1 and MH-Hd1 using RICE6K SNP array

The red line means homozygous exogenous region for one SNP and the blue line means heterozygous region for one SNP.

A


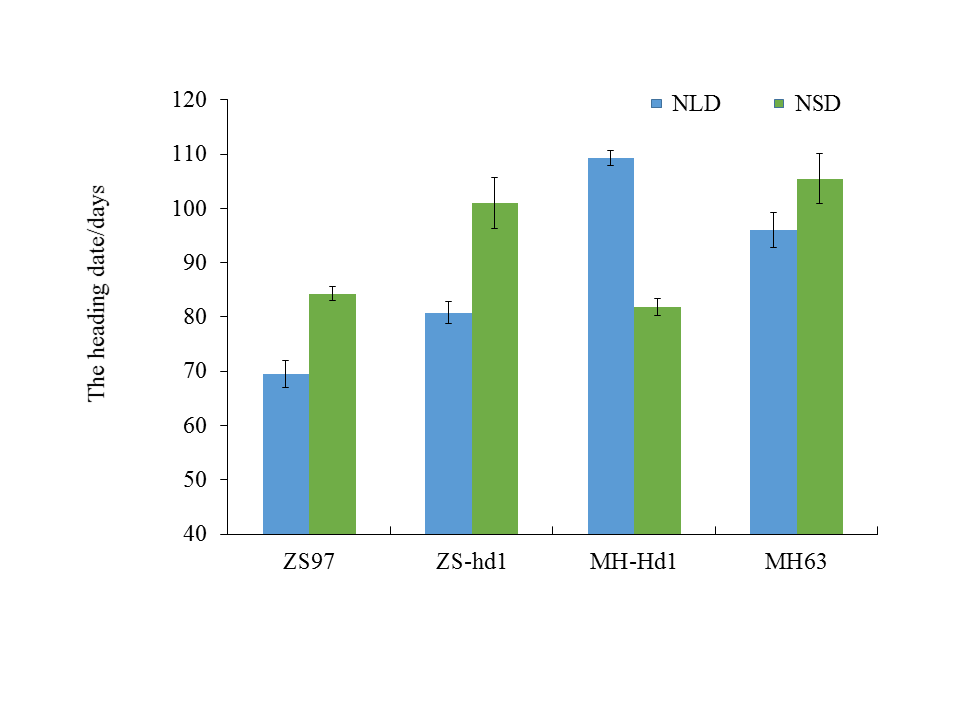


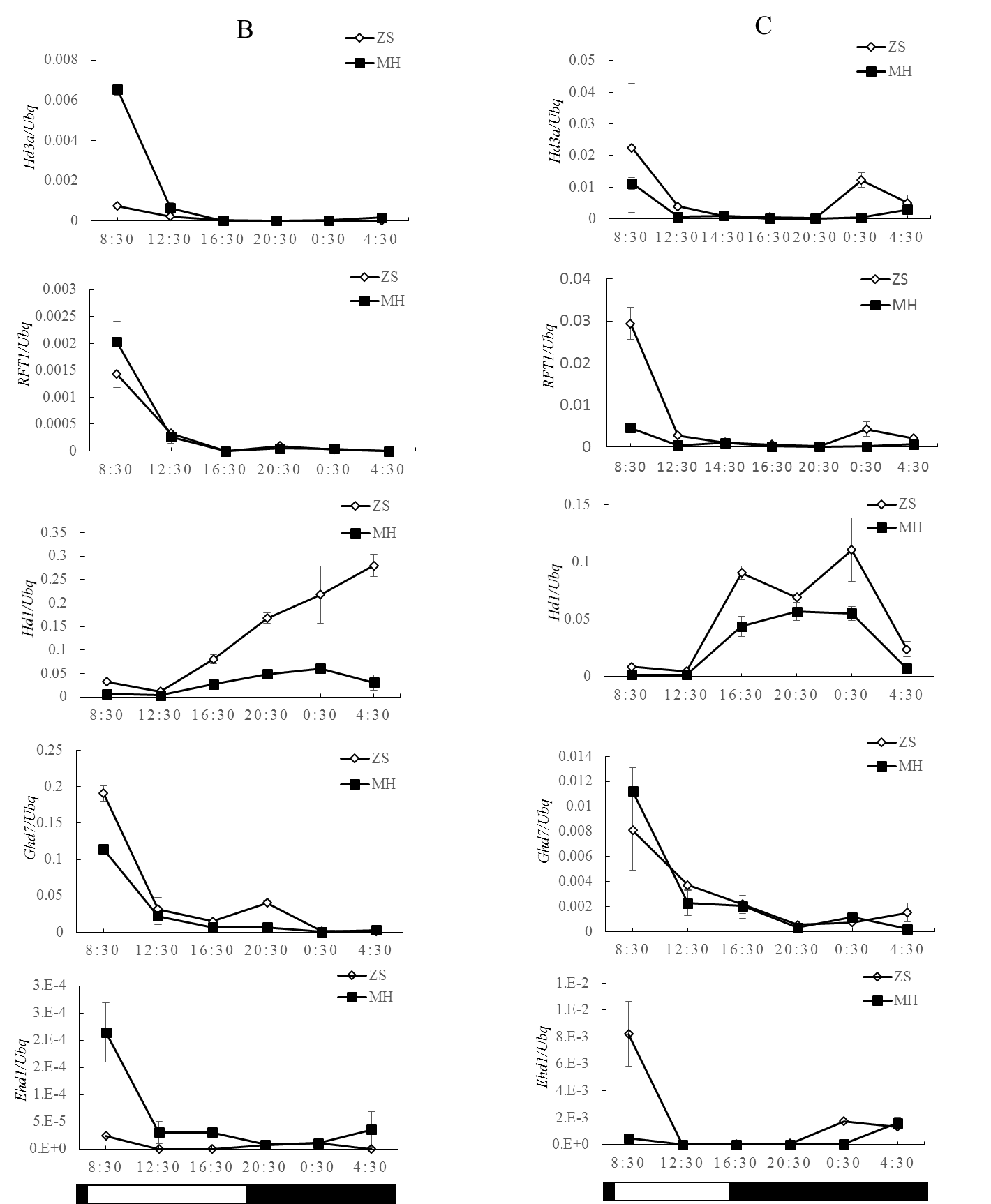


Supplementary Figure 3. Photoperiod sensitivity of *Hd1* in two reciprocal *Hd1* introgression lines and expression patterns of key flowering genes in Minghiu 63 and MH-Hd1

A, the heading date of *Hd1* in Zhenshan 97 and Minghui 63 background under NLD and NSD. NLD, nature long days in Wuhan, Hubei; NSD, nature short days in Lingshui, Hainan.

B and C, the expression of *Hd1*, *Ghd7*, *Ehd1*, *Hd3a*, and *RFT1* in Minghui 63 and MH-Hd1 under LD and SD. ZS and MH represented Minghui 63 carried functional Hd1 and MH63, respectively.


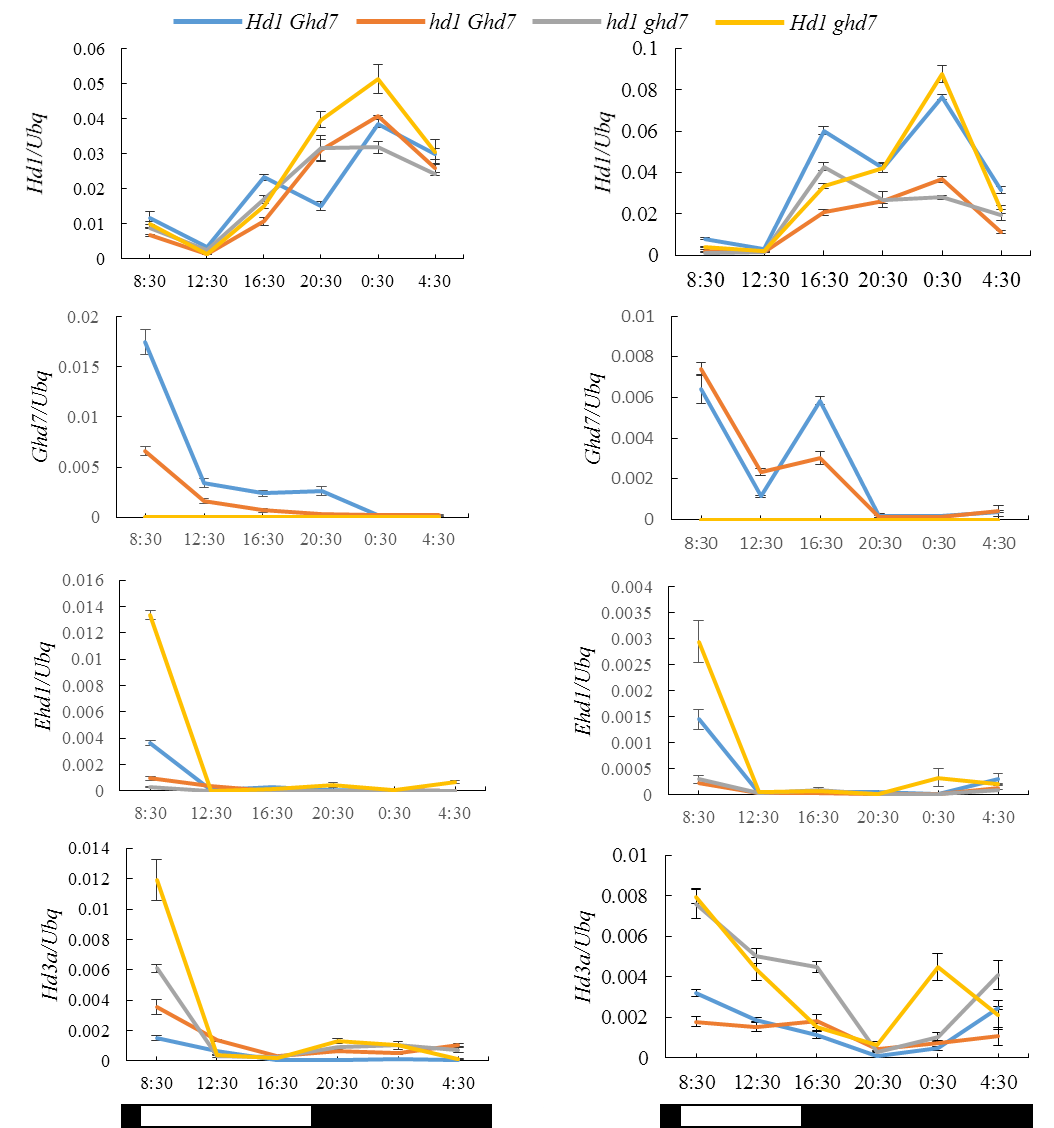


Supplementary Figure 4. The genes expression in 4 combinations of *Ghd7* and *Hd1* in Zhenshan 97 background


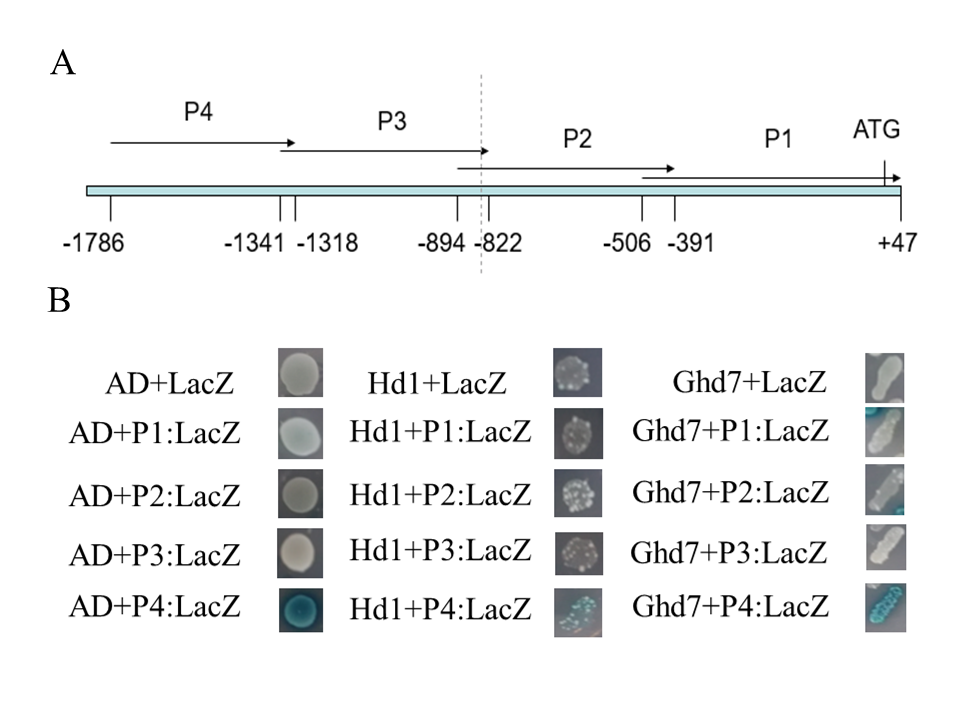


Supplementary Figure 5. The Yeast one-hybrid for Ghd7 and Hd1 binding to the promoter of Ehd1

A, truncate fragments of the promoter of *Ehd1*. B, Hd1 and Ghd7 did not bind to the promoter of *Ehd1* from -1341bp to +47bp, the P4 was activated by AD. “ATG” means the initiation code of *Ehd1*; “AD” means the empty vector of PJG4-5 containing the activation domain of Gal4. “LacZ” means the empty vector of Placzi2μ containing reporter gene LacZ.


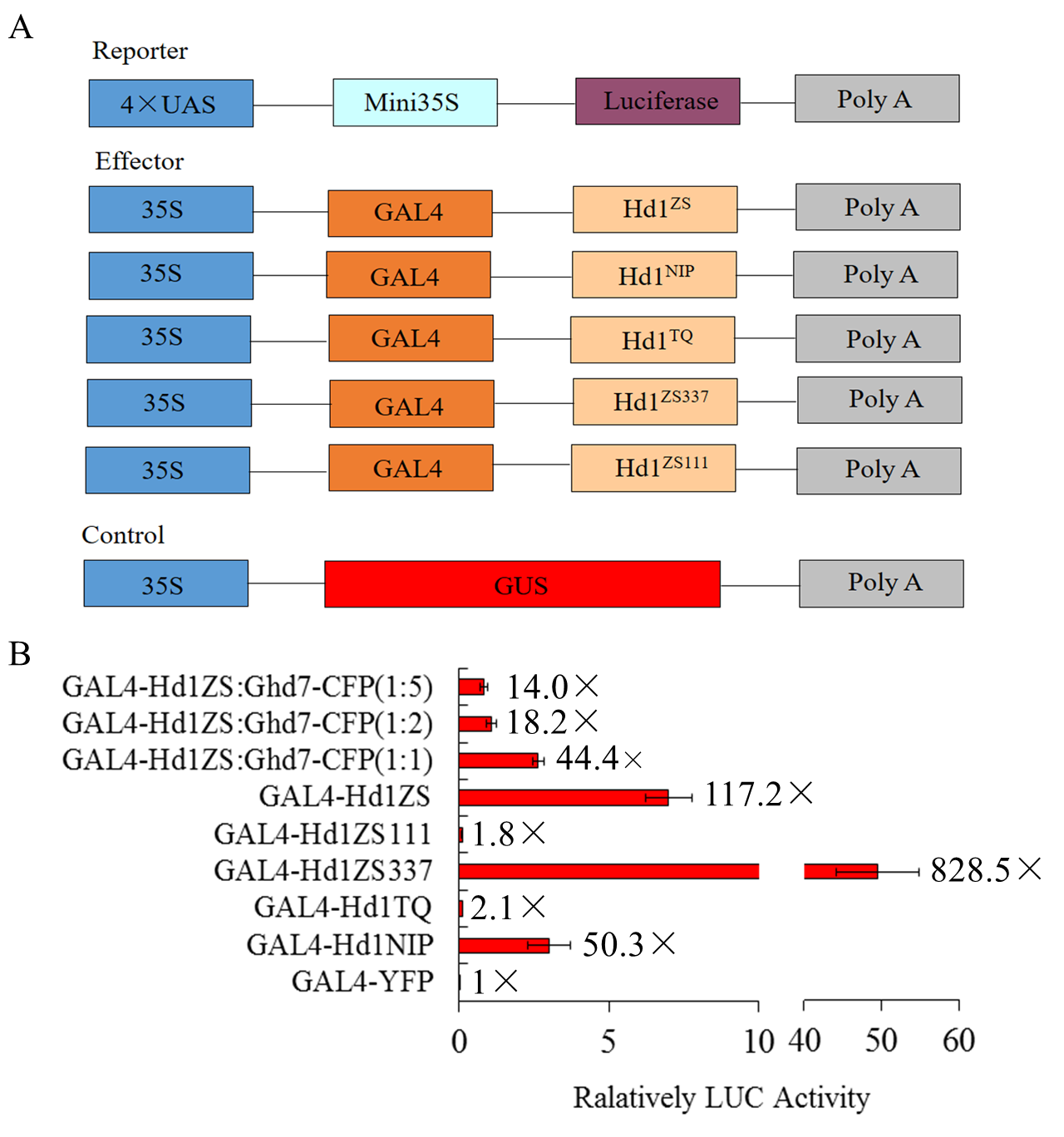


Supplementary Figure 6. Ghd7 represses the transcriptional activation activity of *Hd1*

A, the construction of the vectors for transcriptional activation activity analysis. B, the relative Luciferase activity of transcriptional activation activity analysis. Data are normalized to the internal control 35S:GUS. “ZS”, “NIP”, “TQ” means the coding regions of *Hd1* from Zhenshan 97, Nipponbare and Teqing, respectively. “ZS111” means Zhenshan 97 *Hd1* region coding the amino acids from +1 aa to +111 aa; “ZS337” means Zhenshan 97 *Hd1* region coding the amino acids from +1 aa to +337 aa.
